# Carboxypeptidase A4 promotes cardiomyocyte hypertrophy through activating PI3K-AKT-mTOR signaling

**DOI:** 10.1042/BSR20200669

**Published:** 2020-05-11

**Authors:** Weinian Gao, Na Guo, Shuguang Zhao, Ziying Chen, Wenli Zhang, Fang Yan, Hongjuan Liao, Kui Chi

**Affiliations:** 1 Department of Cardiac Macrovascular Surgery, The Second Hospital of Hebei Medical University, Shijiazhuang 050000, China; 2 Department of Cardiology, Shijiazhuang Translational Chinese Medicine Hospital, Shijiazhuang 050000, China; 3 Department of Vascular Surgery, The Second Hospital of Hebei Medical University, Shijiazhuang 050000, China

**Keywords:** Akt, Cardiac hypertrophy, CPA4, mTOR, PI3K

## Abstract

Carboxypeptidase A4 (CPA4) is a member of the metallocarboxypeptidase family. Current studies have identified the roles of CPA4 in cancer biology and insulin sensitivity. However, the roles of CPA4 in other diseases are not known. In the present study, we investigated the roles of CPA4 in cardiac hypertrophy. The expression of CPA4 was significantly increased in the hypertrophic heart tissues of human patients and isoproterenol (ISO)-induced hypertrophic heart tissues of mice. We next knocked down *Cpa4* with shRNA or overexpressed *Cpa4* using adenovirus in neonatal rat cardiomyocytes and induced cardiomyocyte hypertrophy with ISO. We observed that *Cpa4* overexpression promoted whereas *Cpa4* knockdown reduced ISO-induced growth of cardiomyocyte size and overexpression of hypertrophy marker genes, such as myosin heavy chain β (*β-Mhc*), atrial natriuretic peptide (*Anp*), and brain natriuretic peptide (*Bnp*). Our further mechanism study revealed that the mammalian target of rapamycin (mTOR) signaling was activated by *Cpa4* in cardiomyocytes, which depended on the phosphoinositide 3-kinase (PI3K)-AKT signaling. Besides, we showed that the PI3K-AKT-mTOR signaling was critically involved in the roles of *Cpa4* during cardiomyocyte hypertrophy. Collectively, these results demonstrated that CPA4 is a regulator of cardiac hypertrophy by activating the PI3K-AKT-mTOR signaling, and CPA4 may serve as a promising target for the treatment of hypertrophic cardiac diseases.

## Introduction

In adult mammalian hearts, the proliferative capacity of the cardiomyocytes is limited. Upon injury or increased functional demand, the cardiomyocytes can not proliferate to repair or support the increased demand. Instead, cardiomyocytes can undergo hypertrophic growth, which can compensatorily support the functions of the myocardial tissues [1,2]. Cardiac hypertrophy can be divided into physiological and pathological hypertrophy [3]. Pathological hypertrophy is irreversible and can be induced by hypertension, myocardial infarction, and neuroendocrine factors [3]. The continuous overload of the cardiac tissues and hypertrophic growth leads to arrhythmia, cardiac hypertrophy, and heart failure [1,3]. Therefore, cardiac hypertrophy has been considered the core fundamental mechanism underlying cardiac diseases. Nowadays, cardiovascular diseases are increasing and have been regarded as the first-leading cause of deaths all around the world. Targeting hypertrophy for the treatment of cardiac diseases is a promising strategy [4], which needs a further deep understanding of the mechanisms underlying cardiac hypertrophy.

The hypertrophic growth of cardiomyocytes is regulated by diverse intracellular signalings, such as NAD^+^-dependent protein deacylase Sirtuins, AMP-dependent protein kinase (AMPK), FOXOs, histone deacetylases (HDACs), and the mammalian target of rapamycin (mTOR) [5–9]. For instance, the phosphoinositide 3-kinase (PI3K)-AKT signaling contributes to nearly all kinds of cardiac hypertrophy. PI3K is activated by neuroendocrine factors (e.g., angiotensin II [Ang II], endothelin 1 [ET-1], isoproterenol [ISO]), insulin, insulin-like growth factors, apelin via the G protein-coupled receptors (GPCRs) [10–12]. The phosphorylated PI3K activates the kinase AKT via a phosphoinositide-dependent protein kinase (PDK1)-dependent or independent manner [10]. The kinase Akt can activate its downstream targets via phosphorylation to participate in cell survival, growth, and proliferation [10,13]. One of the central targets of AKT during cardiac hypertrophy is mTOR. mTOR can regulate the activity of ribosomes and promote protein synthesis via phosphorylating the p70 ribosomal protein S6 kinase 1 (S6K1) [13,14]. The mTOR complex is the key modulator for organ and cell size and controls the hypertrophic growth of different kinds of cells, including smooth muscle cells, cardiomyocytes, skeletal muscle cells, and adipocytes [7,13]. Repression of the activity of mTOR by rapamycin can inhibit cardiac hypertrophy in rodents [15,16].

Carboxypeptidase A4 (CPA4) belongs to the metallocarboxypeptidase family, which participates in the regulation of peptide hormone activity and hormone-regulated tissue growth and differentiation [17]. The roles of CPA4 in cancer biology have been widely investigated. For example, the down-regulation of CPA4 represses cancer growth by inhibiting the c‐MYC pathway in human non-small cell lung cancer [18]. CPA4 can also function as the diagnostic and prognostic marker in human breast cancer. CPA4 high-expression is correlated with aggressive phenotype and poor prognosis [19,20]. The role of CPA4 in regulating of adipocytes was also revealed. Fibroblast growth factor-1 (FGF-1) can facilitate adipogenesis by down-regulating CPA4, which is a negative modulator of adipogenesis to participate in local and systemic insulin sensitivity [21]. However, the roles of CPA4 in cardiac hypertrophy remains unknown.

In the present study, we authors aimed to investigate the biological roles of CPA4 in cardiac hypertrophy in humans and rodents. We observed that CPA4 was overexpressed in hypertrophic hearts and cardiomyocytes. The hyperexpression of CPA4 promoted cardiomyocyte hypertrophy through activating the PI3K-AKT-mTOR signaling pathway.

## Materials and methods

### Human patient samples

Human samples (six controls and six cardiac hypertrophy) were obtained at The Second Hospital of Hebei Medical University from May 2016 to Jan 2019. Every patient or control donor signed the written form of informed consent. The study design and experimental protocol were approved by the Ethics Committee of Clinical Research of Hebei Medical University.

### Animal study

For the animal model of cardiac hypertrophy, 8–12 weeks old male C57BL/6 mice were used. Mice were anesthetized with isoflurane and body temperature maintained on a circulating heated waterpad. The cardiac hypertrophy in mouse was induced by subcutaneously chronic infusion of ISO (Sigma; 50 mg/kg/day) with ALZet minipump 2004 for 28 days, as described in previous work [22]. The animal experiments were performed in the Animal Center of Hebei Medical University. The study design and experimental protocol were approved by the Ethics Committee of Animal Research of Hebei Medical University.

### Isolation and culture of neonatal rat cardiomyocytes

To induce *in vitro* cardiomyocyte hypertrophy, neonatal rat cardiomyocytes from 1- to 3-day-old Sprague–Dawley rats were isolated and used using a previously described protocol [23,24]. The rat cardiomyocytes were cultured in DMEM (HyClone) containing 10% fetal bovine serum (FBS, Thermo Fisher), penicillin/streptomycin (1000 U/ml each; Gibco). A total of 100 mM of 5-Bromo-2-deoxyuridine (Sigma) was supplied to repress the growth of cardiac fibroblasts. Forty-eight hours later, the medium was replaced and the cells were used for further experiments.

### *In vitro* model of cardiomyocyte hypertrophy

To induce cardiomyocyte hypertrophy, the cardiomyocytes were starved with 1% FBS for 24 h. Then, the cardiomyocytes were subjected to hypertrophy induction with ISO (50 μM) treatment for 48 h. A-actinin staining was performed to stain and indicate cardiomyocytes, then cell size was analyzed with the ImageJ software. For drug treatment, rapamycin, LY294002, and Ipatasertib were purchased from Selleck and the concentrations were indicated in the figure legends.

### Quantitative real-time PCR

RNA was isolated from fresh tissues and cultured cardiomyocytes using the TRIzol reagent purchased from Thermo Fisher. Two micrograms of RNA was subjected to synthesize the first-strand cDNA by using the cDNA synthesis kit from New England Biolabs. Next, the mRNA expressions of target genes were analyzed with SYBR Green II qRT-PCR kit from QIAGEN on IQ5 (BioRAD). The forward and reverse primers used for quantitative real-time PCR are shown in Table 1.

**Table 1:** Primers used for quantitative real-time PCR in the present study

Gene symbol	Forward primer (5′–3′)	Reverse primer (5′–3′)
*ANP* (h)	AAGAAAGCACACCAACGCAG	GATGGTGACTTCCTCGCCTC
*BNP* (h)	CTGATCCGGTCCATCTTCCT	TGGAAACGTCCGGGTTACAG
*β-MHC* (h)	CCAAGTTCACTCACATCCATCA	AGTGGCAATAAAAGGGGTAGC
*CPA4* (h)	AGGTGGATACTGTTCATTGGGG	TTGCTGATCTCGTCTCCATTTC
*GAPDH* (h)	GGCTGTTGTCATACTTCTCATGG	GGAGCGAGATCCCTCCAAAAT
*Anp* (m)	TCTTCCTCGTCTTGGCCTTT	CCAGGTGGTCTAGCAGGTTC
*Bnp* (m)	TGGGAGGTCACTCCTATCCT	GGCCATTTCCTCCGACTTT
*β-Mhc* (m)	CGGACCTTGGAAGACCAGAT	GACAGCTCCCCATTCTCTGT
*Cpa4* (m)	CCGAGATAAATTCTTTGGGGACC	CCAGACACTGAGCTTTAAGTGG
*Gapdh* (m)	TGTAGACCATGTAGTTGAGGTCA	AGGTCGGTGTGAACGGATTTG
*Anp* (r)	GAAGATGCCGGTAGAAGATGAG	AGAGCCCTCAGTTTGCTTTTC
*Bnp* (r)	CTGGAGACTGGCTAGGACTTC	GGTGCTGCCCCAGATGATT
*Mhc* (r)	GCCCCAAATGCAGCCAT	CGCTCAGTCATGGCGGAT
*Cpa4* (r)	TAGCCTCCGGGGAATGGTAA	CCTCCACTTTTGATCGGCCT
*Gapdh* (r)	TGACAACTCCCTCAAGATTGTCA	GGCATGGACTGTGGTCATGA

Abbreviations: *Anp*, atrial natriuretic peptide; *Bnp*, brain natriuretic peptide; h, human; m, mouse; r, rat; β-Mhc, myosin heavy chain β.

### Western blot

Total protein was extracted from fresh tissues and cultured cardiomyocytes using the RIPA lysis buffer purchased from Millipore. Protease inhibitor cocktail (Roche) was added into the lysis buffer to reduce the degradation of proteins. Then 40 μg of total proteins were separated with SDS/PAGE, followed by membrane transferring and primary antibody incubation at 4°C overnight. Then, the membranes were washed and incubated with secondary antibodies (Servicebio) and expression of proteins was analyzed with ECL kit (Byeotime). Anti-CPA4 and anti-GAPDH antibodies were purchased from Abcam. Anti-pmTOR, anti-mTOR, anti-pS6K1, anti-S6K1, anti-pPI3K, anti-PI3K, anti-pAKT, anti-AKT antibodies were purchased from Cell Signaling Technology.

### Adenovirus-mediated gene silence and overexpression

To silence or overexpress *Cpa4* in rat cardiomyocytes, the adenovirus system was applied. For gene knockdown, short-hairpin RNA (shRNA) targeting rat *Cpa4* (sh*Cpa4*, 5′-GCAAGAAACGGCCAGCCATTT-3′) or control shRNA (shCtrl, 5′-GTTCACCGTAGTTCCGTTC-3′) was cloned into the adenovirus expressing plasmid pAdTrack under the control of U6 promoter. For overexpression *Cpa4* in cardiomyocytes, rat *CPA4* (NM_001109346.2) expressing construct was cloned into the adenovirus expressing plasmid pAdTrack-CMV. The adenovirus packaging plasmids were transfected into HEK293A cells to produce adenovirus using a standard protocol as described previously [25].

### Heart function analysis

The fraction shortening and ejection fraction of control and ISO-infused mice were analyzed as described previously [26,27].

### Protein synthesis assay

Protein synthesis was monitored by incorporation of [^3^H]-leucine into proteins as described previously [28].

### Statistical analysis

All statistical values were shown as mean ± SD of three independent experiments if no other information was stated. To analyze the difference between the two groups, the standard Student’s *t* test was utilized. For analysis of the difference among more than two groups, two-way ANOVA followed by Tukey’s post-hoc test was applied. All the *P*-values less than 0.05 were considered significant. All statistical analyses were performed with the GraphPad Prism software version 8.0.

## Results

### The expression of CPA4 is up-regulated in human and mouse hypertrophic hearts

**Figure 1:**
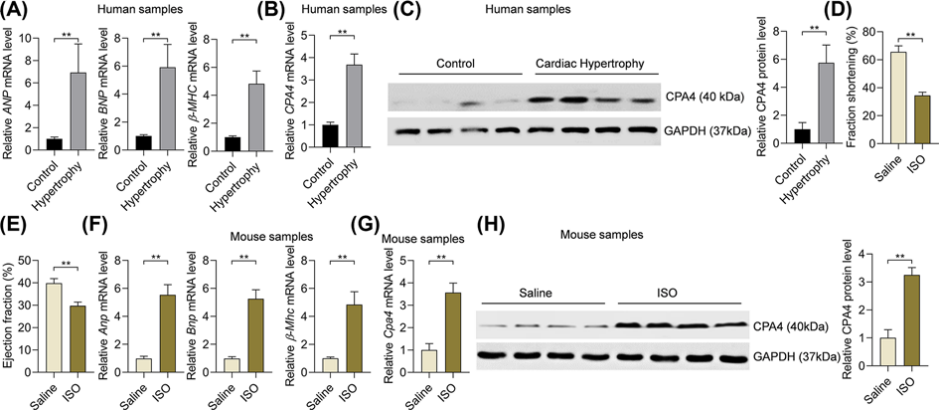
CPA4 is overexpressed in hypertrophic hearts of human and mouse (**A**) mRNA levels of hypertrophy-associated marker genes in control (*n*=6) and hypertrophic hearts (*n*=6) in humans. The mRNA levels were analyzed by qRT-PCR. **P<0.01. (**B**) mRNA level of *CPA4* in control (*n*=6) and hypertrophic hearts (*n*=6) in humans. The mRNA level was analyzed by qRT-PCR. **P<0.01. (**C**) The protein level of CPA4 in control (*n*=5) and hypertrophic hearts (*n*=56) in humans. The protein level of CPA4 was analyzed by Western blot. **P<0.01. (**D**) Fractional shortening of mice with cardiac hypertrophy. Cardiac hypertrophy was induced in male C57BL/6 mice with ISO (50 mg/kg, subcutaneously) treatment continuously for 28 days. *n*=5, **P<0.01. (**E**) Ejection fraction of mice with cardiac hypertrophy. *n*=5, **P<0.01. (**F**) mRNA levels of hypertrophy-associated marker genes in control (*n*=5) and ISO-induced hypertrophic hearts (*n*=5) in mice. **P<0.01. (**G**) mRNA level of *Cpa4* in control (*n*=5) and ISO-induced hypertrophic hearts (*n*=5) in mice. **P<0.01. (**H**) The protein level of CPA4 in control (*n*=5) and ISO-induced hypertrophic hearts (*n*=5) in mice. The statistical analysis was performed with Student’s *t* test.

CPA4 is a member of the metallocarboxypeptidase family. However, the functional involvement of CPA4 in the cardiovascular system is not clear. Here we attempted to determine the roles of CPA4 in cardiac hypertrophy in humans and rodents. To this purpose, we initially monitored the expression changes in CPA4 during cardiac hypertrophy. We collected six samples of hypertrophic hearts and six control heart tissues, and the hypertrophic growth of human hearts varied with the up-regulation of hypertrophy marker genes, such as atrial natriuretic peptide (*ANP*), brain natriuretic peptide (*BNP*), and myosin heavy chain β (*β-MHC*) (Figure 1A). Next, we tested the mRNA and protein levels of *CPA4* with qRT-PCR and Western blot assays, respectively. The results significantly showed that the expression levels of both mRNA and protein of *CPA4* were much higher in hypertrophic hearts compared with the controls (Figure 1B,C).

Next, we tested whether the up-regulation of CPA4 in hypertrophic hearts was conserved across species. Cardiac hypertrophy was induced in C57BL/6 mice by subcutaneous treatment of ISO (50 mg/kg/day) for continuous 28 days using a protocol reported previously [22]. ISO treatment induced the decrease in fraction shortening and ejection fraction in mice (Figure 1D,E). The hypertrophic growth of mouse hearts was coupled with the overexpression of hypertrophy-associated fetal genes (*Anp, Bnp*, and *β-Mhc*) (Figure 1F). Similar to the pattern in humans, the mRNA and protein levels of *Cpa4* was significantly higher in ISO-induced hypertrophic hearts compared with the control hearts (Figure 1G,H).

### CPA4 is involved in the development of cardiomyocyte hypertrophy

**Figure 2:**
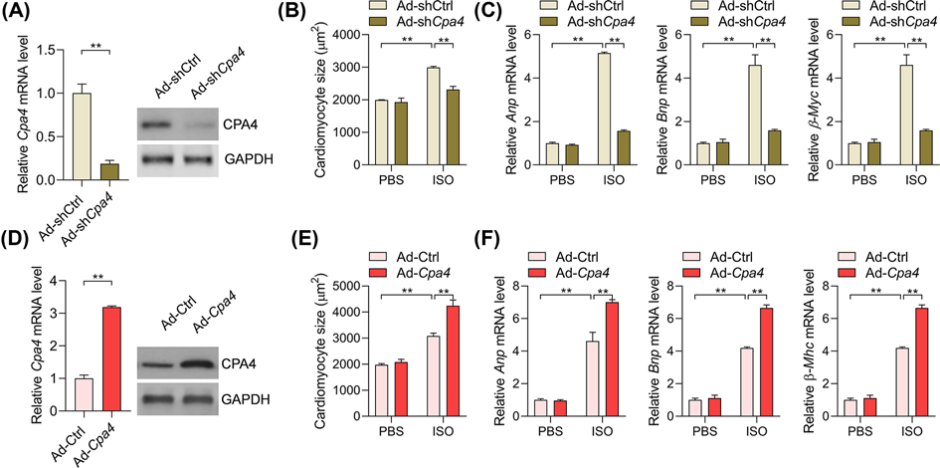
CPA4 promotes the ISO-induced cardiomyocyte hypertrophy (**A**) shRNA-mediated knockdown of *Cpa4* in rat cardiomyocytes. The rat cardiomyocytes were infected with adenovirus expressing shCtrl (Ad-shCtrl) or sh*Cpa4* (Ad-sh*Cpa4*), mRNA, and protein levels were analyzed by qRT-PCR and Western blot 2 days later (*n*=3). **P<0.01. (**B**) *Cpa4* deficiency represses ISO-induced growth in cardiomyocytes. The rat cardiomyocytes were infected with Ad-shCtrl or Ad-sh*Cpa4* for 24 h, followed by ISO treatment (50 μM) for additional 48 h to induce cardiomyocyte hypertrophy. ImageJ was used to quantify the cardiomyocytes size (*n*=3). **P<0.01. (**C**) *Cpa4* deficiency represses ISO-induced overexpression of hypertrophy-associated genes. The rat cardiomyocytes were treated as in (B), followed by an analysis of target genes with qRT-PCR (*n*=3). **P<0.01. (**D**) Adenovirus-mediated overexpression of *Cpa4* in rat cardiomyocytes. The rat cardiomyocytes were infected with adenovirus carrying rat *Cpa4* (Ad-*Cpa4*) or control adenovirus (Ad-Ctrl) for two days, followed by the analysis of mRNA and protein levels with qRT-PCR and Western blot (*n*=3). **P<0.01. (**E**) *Cpa4* overexpression enhances ISO-induced growth in cardiomyocytes. The rat cardiomyocytes were infected with Ad-Ctrl or Ad-*Cpa4* for 24 h, followed by ISO treatment (50 μM) for additional 48 h to induce cardiomyocyte hypertrophy. ImageJ was used to quantify the cardiomyocytes size (*n*=3). **P<0.01. (**F**) *Cpa4* overexpression facilitates ISO-induced overexpression of hypertrophy-associated genes. The rat cardiomyocytes were treated as in (E), followed by an analysis of mRNA and protein levels with qRT-PCR and Western blot (*n*=3). **P<0.01. In (A,D), the statistical analysis was performed with Student’s *t* test. In (B,C,E,F), the statistical analysis was performed with two-way ANOVA followed by Tukey’s *post-hoc* test.

Since CPA4 is overexpressed in hypertrophic myocardial tissues in humans and rodents, CPA4 may regulate the progress of cardiomyocyte hypertrophy. To monitor the function of CPA4, *loss-of-function* (gene silence) and *gain-of-function* (gene overexpression) strategies were carried out by using the adenovirus system. Adenovirus-mediated shRNA targeting *Cpa4* (sh*Cpa4*) significantly down-regulated the expression levels of Cpa4 in rat cardiomyocytes (Figure 2A). Next, we used an *in vitro* model of cardiomyocyte hypertrophy by treating neonatal rat cardiomyocytes with ISO for 48 h. ISO treatment remarkedly induced the increase in cardiomyocyte size and triggered the hyperexpression of hypertrophic fetal genes (*Anp, Bnp*, and *β-Mhc*) (Figure 2B,C). Notably, *Cpa4* knockdown repressed ISO-induced hypertrophic growth of rat cardiomyocytes (Figure 2B,C). *Cpa4* was also overexpressed in rat cardiomyocytes using the adenovirus system (Figure 2D). By contrast, *Cpa4* overexpression can facilitate the increase in the size of cardiomyocytes and overexpression of hypertrophic fetal genes (Figure 2E,F). Collectively, these findings demonstrated that CPA4 promotes cardiomyocyte hypertrophy.

### CPA4 promotes mTOR activation to participate in cardiomyocyte hypertrophy

**Figure 3:**
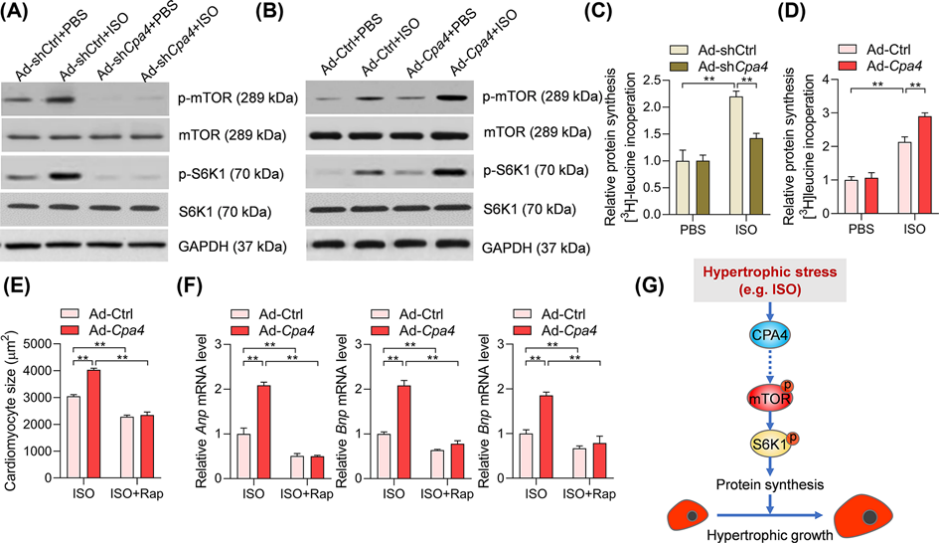
CPA4 promotes mTOR activation to promote cardiomyocyte hypertrophy (**A**) *Cpa4* deficiency reduces ISO-induced activation of the mTOR-S6K1 signaling pathway. The rat cardiomyocytes were infected with Ad-shCtrl or Ad-sh*Cpa4* for 24 h, followed by ISO treatment (50 μM) for ditional 24 h. The protein levels of target protein were analyzed by Western blot. (**B**) *Cpa4* overexpression enhances ISO-induced activation of the mTOR-S6K1 signaling pathway. The rat cardiomyocytes were infected with Ad-Ctrl or Ad-*Cpa4* for 24 h, followed by ISO treatment (50 μM) for an additional 24 h. The protein levels of target protein were analyzed by Western blot. (**C**) Effects of CPA4 knockdown on protein synthesis in ISO-treated cardiomyocytes (*n*=3). **P<0.01. (**D**) Effects of CPA4 overexpression on protein synthesis in ISO-treated cardiomyocytes (*n*=3). **P<0.01. (**E**) Inhibition of mTOR with rapamycin blocks CPA4-mediated increase in cardiomyocyte size. The cardiomyocytes were infected with Ad-*Cpa4* for 24 h, and then the cells were treated with ISO (50 μM) and rapamycin (100 nM) was added to inhibit mTOR for 48 h. ImageJ was used to quantify cell size (*n*=3). **P<0.01. (**F**) Inhibition of mTOR with rapamycin blocks CPA4-mediated overexpression of hypertrophic marker genes. The rat cardiomyocytes were treated as in (C), then analysis of mRNA levels of target genes was carried out with qRT-PCR (*n*=3). **P<0.01. (**G**) Illustrator showing CPA4 activates mTOR-S6K1 signaling to promote cardiomyocyte hypertrophy. The statistical analysis was performed with two-way ANOVA followed by Tukey’s *post-hoc* test.

Next, we investigated the underlying biological mechanisms. mTOR is a pivotal regulator for protein synthesis and cardiac hypertrophy via activating the ribosomal protein S6K1 [14]. We hypothesized that CPA4 may regulate cardiomyocyte hypertrophy via the mTOR-SK61 signaling pathway. To test this hypothesis, we performed Western blot to analyze the activation of mTOR-S6K1 signaling in cardiomyocytes. ISO treatment increased the phosphorylation of mTOR and S6K1 in rat cardiomyocytes. Remarkably, adenovirus-mediated *Cpa4* knockdown repressed ISO-induced increase in phosphorylation of mTOR and S6K1 in rat cardiomyocytes (Figure 3A). By contrast, *Cpa4* overexpression facilitated the increase in phosphorylation of mTOR and S6K1 in cardiomyocytes treated by ISO (Figure 3B). Therefore, CPA4 participates in ISO-induced activation of mTOR-S6K1 signaling during cardiomyocyte hypertrophy. mTOR-S6K1 is an important regulator for protein synthesis during cardiac hypertrophy. We also analyzed the effects of CPA4 on protein synthesis. The results showed that CPA4 knockdown repressed while CPA4 overexpression promoted ISO-induced protein synthesis in cardiomyocytes (Figure 3C,D). Besides, we explored whether the activation of mTOR-S6K1 mediated by CPA4 overexpression contributed to the function of CPA4. We treated the cardiomyocytes with mTOR inhibitor rapamycin. Rapamycin treatment significantly repressed cardiomyocyte size and marker gene expressions in ISO-treated cardiomyocytes (Figure 3E,F). Of note, the pro-hypertrophic roles of CPA4 were repressed remarkedly by rapamycin (Figure 3E,F). Therefore, these results implicated that CPA4 activated mTOR-S6K1 signaling, which was essentially involved in CPA4-mediated pro-hypertrophic functions (Figure 3G).

### CPA4 promotes the activation of PDK1 to regulate mTOR and cardiomyocyte hypertrophy

**Figure 4:**
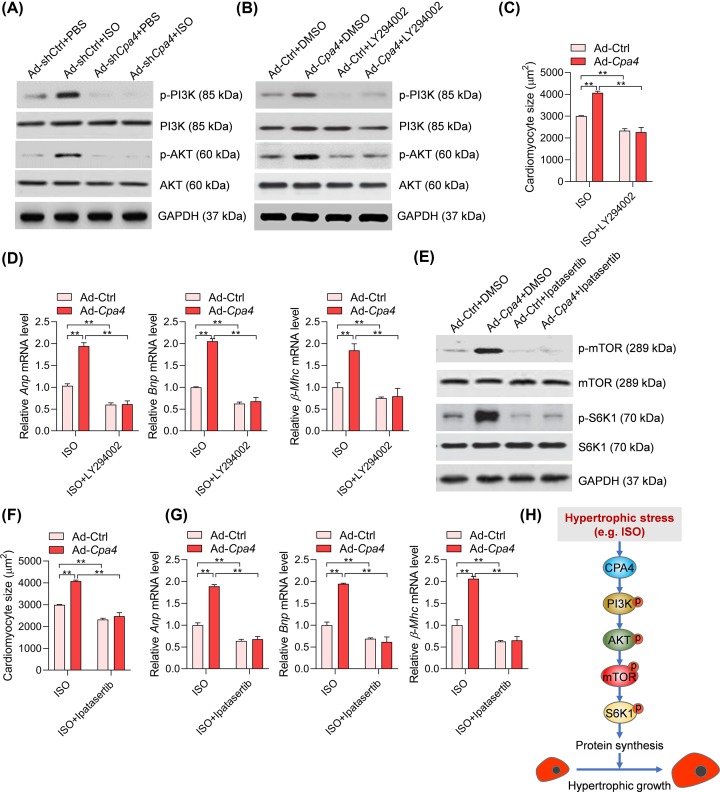
PI3K-AKT is essential for CPA4 function during cardiomyocyte hypertrophy (**A**) *Cpa4* deficiency reduces the PI3K-AKT signaling pathway. The rat cardiomyocytes were infected with Ad-shCtrl or Ad-sh*Cpa4* for 24 h, followed by ISO treatment (50 μM) for an additional 24 h. The protein levels of target protein were analyzed by Western blot. (**B**) Inhibition of PI3K blocks the effects of CPA4-mediated activation of mTOR-S6K1 signaling. The cardiomyocytes were infected with Ad-*Cpa4* for 24 h, and then the cells were treated with ISO (50 μM) and PI3K inhibitor (LY294002, 1 μM) was added to inhibit PI3K for 24 h. The protein levels of target protein were analyzed by Western blot. (**C**) Inhibition of PI3K blocks CPA4-mediated increase in cardiomyocytes. The cardiomyocytes were infected with Ad-*Cpa4* for 24 h, and then the cells were treated with ISO (50 μM) and PI3K inhibitor (LY294002, 1 μM) was added to inhibit PI3K for 24 h. Cardiomyocyte size was quantified with ImageJ (*n*=3). **P<0.01. (**D**) Inhibition of PI3K blocks CPA4-mediated overexpression of hypertrophic marker genes. The rat cardiomyocytes were treated as in (C), then analysis of mRNA levels of target genes was carried out with qRT-PCR (*n*=3). **P<0.01. (**E**) Inhibition of AKT blocks CPA4-mediated activation of mTOR-S6K1 signaling. The cardiomyocytes were infected with Ad-*Cpa4* for 24 h, and then the cells were treated with ISO (50 μM) and AKT inhibitor (Ipatasertib, 1 μM) was added to inhibit AKT for 24 h. The protein levels of target protein were analyzed by Western blot. (**F**) Inhibition of AKT blocks CPA4-mediated increase in cardiomyocytes. The cardiomyocytes were infected with Ad-*Cpa4* for 24 h, and then the cells were treated with ISO (50 μM) and AKT inhibitor (Ipatasertib, 1 μM) was added to inhibit AKT for 48 h. (**G**) Inhibition of AKT blocks CPA4-mediated overexpression of hypertrophic marker genes. The rat cardiomyocytes were treated as in (F), then analysis of mRNA levels of target genes was carried out with qRT-PCR (*n*=3). **P<0.01. (**H**) Illustrator showing CPA4 activates PI3K-AKT-mTOR-S6K1 signaling to promote cardiomyocyte hypertrophy. The statistical analysis was performed with two-way ANOVA and Tukey’s *post-hoc* test.

Then, we attempted to study how CPA4 regulates mTOR signaling to promote cardiomyocyte hypertrophy. A previous study showed that CPA4 activated the kinase AKT in cancer cells [18]. Indeed, we also observed that the ISO-induced activation of the PI3K-AKT signaling pathway was repressed by *Cpa4* deficiency (Figure 4A). As thus, we hypothesized that CPA4 may regulate the PI3K-AKT signaling to promote the activation of the mTOR-S6K1 pathway and cardiomyocyte hypertrophy. To test the hypothesis, we first repressed the activation of PI3K with LY294002 and monitored the activation of the mTOR-S6K1 pathway. LY294002 treatment significantly repressed CPA4-mediated hyperactivation of the mTOR-S6K1 pathway (Figure 4B). Importantly, LY294002 also blocked the effects of CPA4 overexpression on cardiomyocyte size and expression of hypertrophic fetal genes (Figure 4C,D). Similarly, we also repressed AKT with its inhibitor Ipatasertib. Ipatasertib treatment blocked CPA4-mediated hyperactivation of the mTOR-S6K1 pathway, subsequently repressed CPA4 function in regulating cardiomyocyte size and hypertrophic marker gene expression in ISO-induced cardiomyocyte hypertrophy (Figure 4E–G). Taken together, these findings demonstrated that CPA4 activated the PI3K-AKT signaling to promote the activation of the mTOR-S6K1 pathway and subsequent hypertrophy of cardiomyocytes (Figure 4H).

## Discussion

CPA4 is one of the members of the metallocarboxypeptidase family and it participates in cancer biology. Here we identified the roles of CPA4 in regulating PI3K-AKT-mTOR signaling and promotes cardiac hypertrophy. The expression of CPA4 was overexpressed in human and mouse cardiac hypertrophy. Gene silencing and gene overexpression experiments demonstrated that CPA4 promoted ISO-induced hypertrophy of cardiomyocytes. Further biological mechanism studies revealed that CPA4 promoted ISO-induced activation of the mTOR-S6K1 pathway through activating PI3K-AKT signaling.

CPA4 is a metallocarboxypeptidase that regulates peptide hormone activity and hormone-regulated tissue growth and differentiation [15]. The physiological and pathological roles of CPA4 are not fully understood. Most studies on CPA4 focus on its roles in cancer biology. Previous studies have reported the critical involvement of CPA4 in non-small lung cancer [18], breast cancer [19], hepatocellular carcinoma [29], and gastric cancer [30]. Besides, the role of CPA4 in regulating adipogenesis and insulin sensitivity was also observed in adipose tissues [21]. However, the other roles of CPA4 remain unknown. Here we demonstrated that CPA4 was an important regulator for cardiac hypertrophy across species. CPA4 mRNA and protein levels were up-regulated in hypertrophic myocardial tissues in humans and mice. *Gain-of-function* and *loss-of-function* experiments collectively demonstrated that CPA4 promoted ISO-induced cardiomyocyte hypertrophy. Therefore, we identified the pro-hypertrophic roles of CPA4, which implicating that CPA4 is a critical regulator for the cardiovascular system. Since CPA4 was associated with adipogenesis and insulin sensitivity [21], CPA4 may also participate in obesity-associated and diabetic cardiomyopathies.

The mTOR-S6K1 is critically involved in *de novo* protein synthesis and controls cellular and organ size [14]. During cardiac hypertrophy, the mTOR-S6K1 pathway is activated and promotes cardiomyocyte hypertrophy, cardiac remodeling, and heart failure [11,14]. Besides, previous studies have demonstrated that mTOR inhibitor rapamycin is a promising drug for the treatment of cardiac hypertrophy in rodents [11,13–16]. The upstream regulators of the mTOR-S6K1 signaling pathway are not fully identified. Herein the present study, we identified CPA4 as an indirect upstream activator for the mTOR-S6K1 signaling pathway and activated mTOR during cardiac hypertrophy. Also, we observed that rapamycin treatment blocked the function of CPA4 in cardiomyocyte hypertrophy, implicating that mTOR is essential for CPA4 to show its biological functions.

PI3K-AKT signaling is activated by neuroendocrine factors, insulin, and insulin-like growth factors [10]. The activated AKT can promote the activation of mTOR through phosphorylating and inhibiting mTOR upstream repressor tuberous sclerosis complex (TSC)1/2 complex [14]. Indeed, we observed that CPA4 contributed to ISO-induced activation of PI3K-AKT signaling. The previous study showed that CPA4 deficiency suppressed AKT activation [18], which was consistent with our results. And we showed that PI3K mediated the effects of CPA4 on the kinase AKT. Importantly, we observed that inhibition of PI3K with LY294002 or inhibition of AKT with Ipatasertib blocked the activation effects of CPA4 on the mTOR-S6K1 signaling pathways. Also, the roles of CPA4 was blocked by either inhibition of PI3K with LY294002 or inhibition of AKT with Ipatasertib. Therefore, CPA4 activated the mTOR-S6K1 pathway and promoted cardiomyocyte hypertrophy via up-regulating the activation of PI3K-AKT signaling. Since the PI3K-AKT plays central roles in regulating insulin pathways [6,21], CPA4 may serve as an important regulator for insulin response. In the present study, we did not explore the mechanism underlying the CPA4-mediated regulation of PI3K. This may be one of the limitations of the present study. A previous report showed that CPA4 activated STAT3 in cancer cells [31]. Besides, STAT3 was known to activate PI3K signaling in an RAS-dependent manner [32–34]. Therefore, one potential mechanism by which CPA4 activated PI3K was the activation of STAT3-RAS signaling. But further study is needed to support the hypothesis.

In summary, using human samples and animal and cell models, we demonstrated the pro-hypertrophic roles of CPA4. CPA4 facilitates the development of cardiac hypertrophy by activating the PI3K-AKT-mTOR signaling pathway. Therefore, we identified CPA4 as a new upstream regulator of the mTOR pathway and CPA4 may serve as a potential therapeutic target for cardiac hypertrophy.

## Abbreviations

Anp: atrial natriuretic peptide
Bnp: brain natriuretic peptide
CPA4: carboxypeptidase A4
ISO: isoproterenol
mTOR: mammalian target of rapamycin
PDK1: phosphoinositide-dependent protein kinase
PI3K: phosphoinositide 3-kinase
shRNA: short-hairpin RNA
S6K1: p70 ribosomal protein S6 kinase 1
β-Mhc: myosin heavy chain β
